# Determining if T cell antigens are naturally processed and presented on HLA class I molecules

**DOI:** 10.1186/s12865-022-00478-4

**Published:** 2022-02-11

**Authors:** Jay Friedman, Sreenivasulu Gunti, Maxwell Lee, Ke Bai, Christian Hinrichs, Clint T. Allen

**Affiliations:** 1 Section on Translational Tumor Immunology, National Institute on Deafness and Other Communication Disorders, National Institutes of Health, Building 10, Room 7N240C, Bethesda, MD 20892 USA; 2 Rutgers Cancer Center at Rutgers, The State University of New Jersey, New Brunswick, NJ USA

**Keywords:** HLA restriction, Antigen presenting cells, Minimal epitope, Electroporation, T cell responses

## Abstract

**Background:**

Determining T cell responses to naturally processed and presented antigens is a critical immune correlate to determine efficacy of an investigational immunotherapeutic in clinical trials. In most cases, minimal epitopes and HLA restriction elements are unknown.

**Results:**

Here, we detail the experimental use of ex vivo expanded autologous B cells as antigen presenting cells to overcome the limitation of unknown HLA restriction, and the use of electroporated full length mRNA encoding full length parental proteins to ensure that any observed T cell responses are specific for antigens that are naturally processed and presented.

**Conclusions:**

This technique can serve as useful experimental approach to determine the induction or enhancement of specific responses to naturally processed and presented antigens on HLA class I molecules in peripheral blood or tumor infiltrating T cells.

## Background

Most current Food and Drug Administration-approved and investigational immunotherapies for cancer are designed to augment, activate or replace T cell immunity. Examples include immune checkpoint blockade to unblock the activity of existing T cell clones, therapeutic vaccines to activate new or expand existing T cell clones, and adoptive transfer of T cells to replace the existing T-lymphocyte repertoire [1–3]. The ability to experimentally detect antigen-specific responses in T cells from the periphery or tumor is a critical readout to demonstrate the efficacy of these treatments. However, especially when T cells being studied are isolated from patients with non-virally driven malignancies, the minimal antigenic epitope is often unknown.

The use of antigen presenting cells (APCs) loaded with overlapping peptides spanning the candidate protein from which the minimal epitope may be derived is a common approach to assess such T cell responses. The exogenous application of peptides 15 amino acids in length (15mer) overlapping by 11 amino acids can induce CD4+ and CD8+ T lymphocyte responses [4, 5]. This indicates that APCs can cross present human leukocyte antigen (HLA) class I-restricted antigens after endocytosis of exogenous peptides. Surface loading of antigenic peptide can also occur in select conditions [6, 7]. Use of 15mer peptides also allows the determination of putative minimal epitopes responsible for observed T cell responses. However, this approach may bypass one or more steps in the natural processing and presentation of antigen derived from an endogenous intracellular full-length protein, potentially leading to falsely positive results [8]. As tumor-associated antigens or tumor-specific antigens presented via HLA class I molecules are derived from endogenous intracellular proteins within cancer cells, T cells responses are clinically relevant only if they are specific for antigen that is naturally processed and presented from the parental protein. Efficient techniques to screen if an observed antigen-specific T cells response is against an antigen that is naturally processed and presented form the parental protein are needed.

**Fig. 1:**
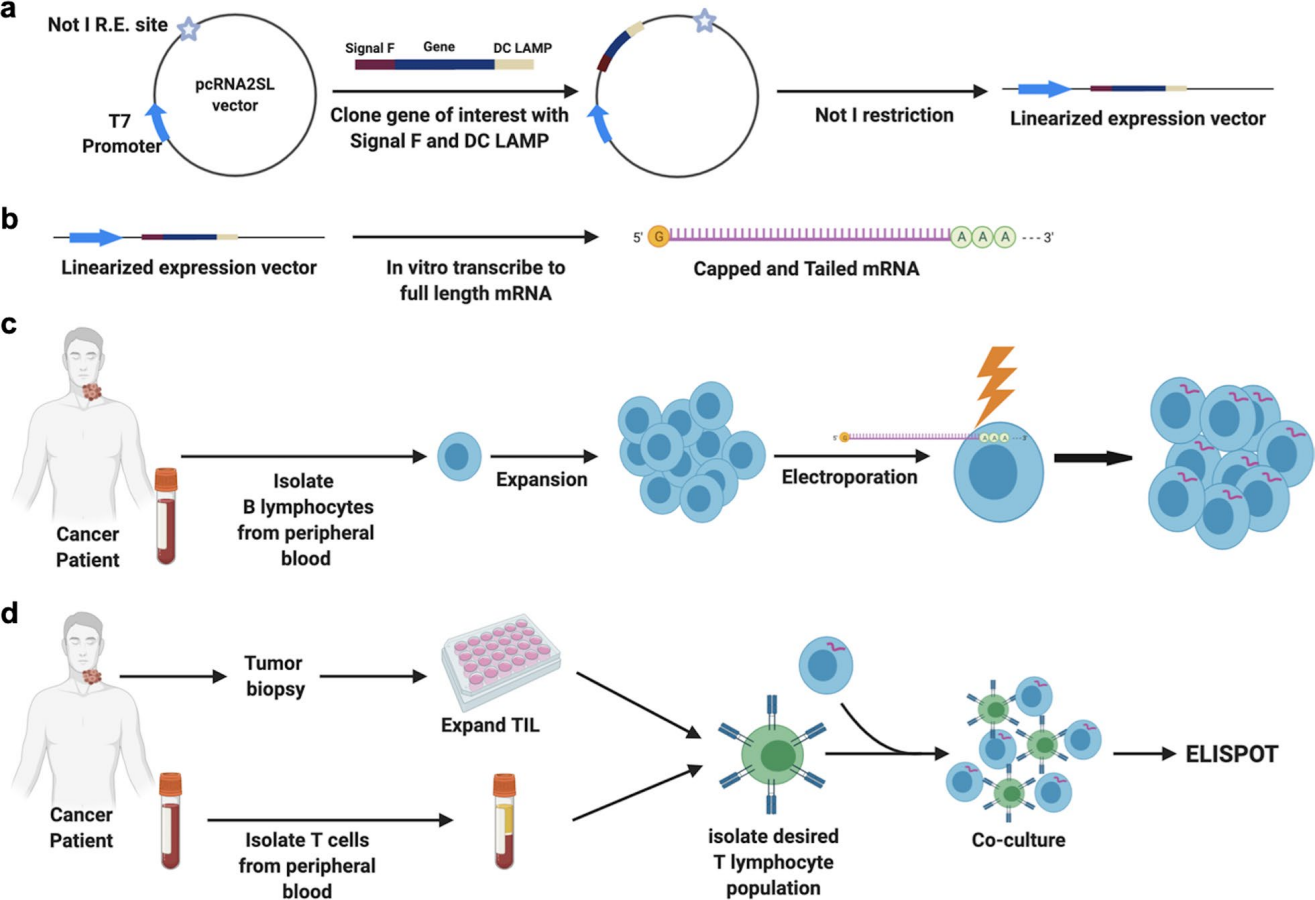
Schematic overview of determining antigen-specific T cell responses using electroporated B cells electroporated with full-length mRNA. **a** pcRNA2SL vector engineered to express a gene of interest is linearized through restriction digestion; **b** linearized expression plasmid is transcribed into full length 5′ capped and 3′ polyA tailed mRNA through in vitro transcription with a commercially available kit; **c** peripheral blood B cells are isolated from patient material through magnetic selection and full-length mRNA is introduced via electroporation; **d** peripheral blood or tumor infiltrating T cells are co-incubated with autologous B cells loaded with full-length mRNA and antigen-specific responses are detected. *R.E.* restriction element, *TIL* tumor infiltrating lymphocytes

Introduction of nucleic acid encoding a whole protein into APCs to mimic an endogenous intracellular protein is one approach to ensure experimentally observed T cell responses are specific for a naturally processed and presented antigen. Here we describe a validated method of electroporating full-length mRNA into autologous APCs for use in T cell co-cultures and assessment of antigen-specific T lymphocyte responses. Autologous B cells, which can be readily expanded in culture for experimental use, were used as APCs. T cells engineered to express a previously characterized T cell receptor (TCR) targeting HPV 16 E7_11–19_ presented via HLA-A*02 were utilized as effector T cells in validation experiments. A schematic overview of the process is detailed in Fig. 1. This approach does not assist in the identification of minimal epitopes or HLA restriction elements for a given T cell response but does ensure that an observed T cell responses is specific for an epitope that is naturally processed and presented from a candidate endogenous full-length parental protein. Such approaches may be useful when tracking the development of antigen-specific T cell responses following immunotherapy or in the process of discovering potentially therapeutic TCRs.

## Results

### B cells used for APCs efficiently expanded in culture

**Fig. 2:**
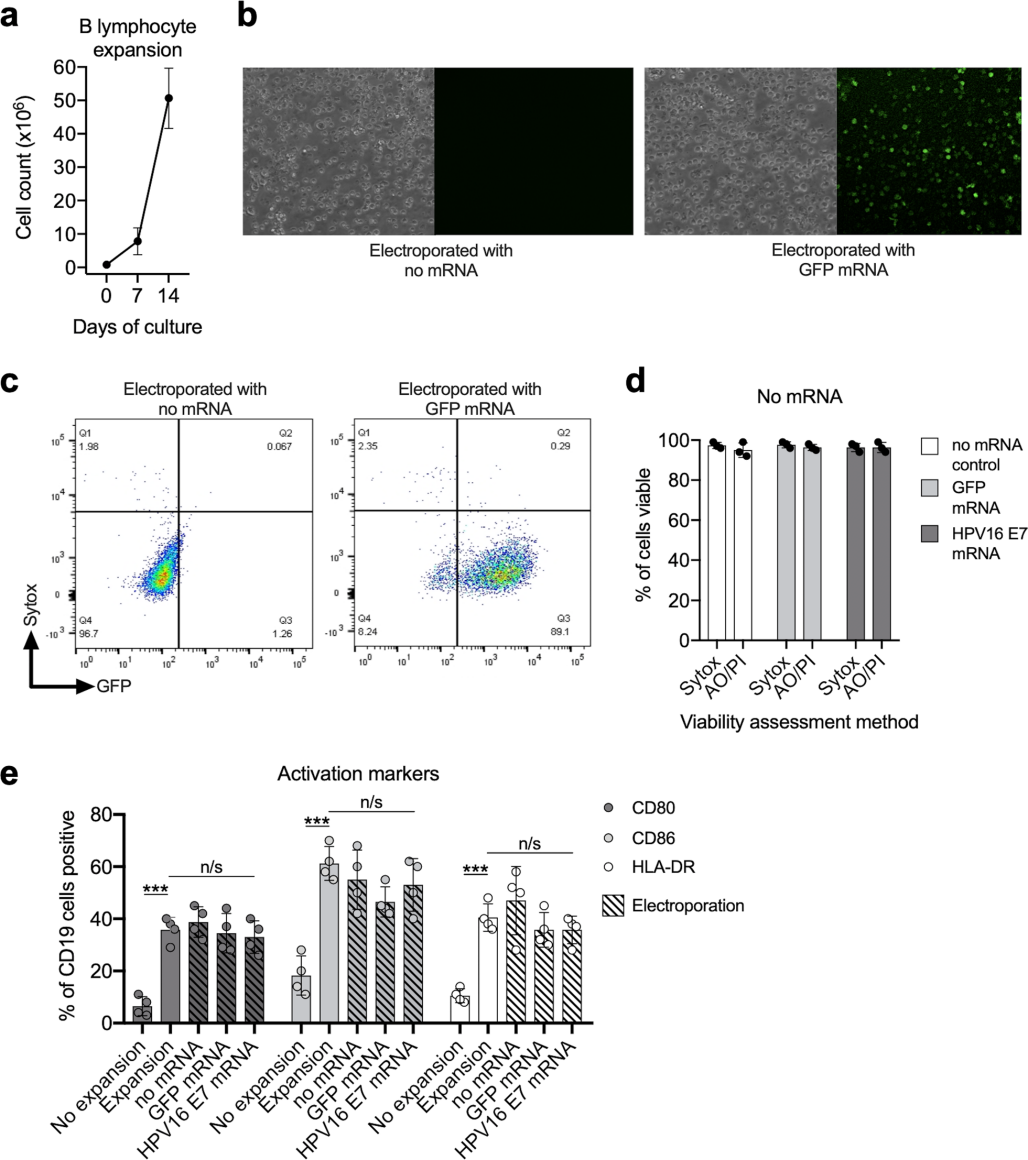
B cells are readily expanded in culture and electroporated with full-length mRNA. **a** Cell counts of magnetically isolated B cells co-cultured with NIH3T3-CD40L feeder cells and IL-4, error bars represent standard deviation of three independent experiments; **b** phase contrast and immunofluorescent photomicrographs of ex vivo expanded B cells electroporated without or with mRNA encoding GFP. Images acquired 2 h after electroporation; **c** flow cytometric analysis of ex vivo expanded B cells electroporated with mRNA encoding GFP for fluorescent signal; **d** viability of B cells electroporated without mRNA (control), with GFP mRNA or with HPV16 mRNA was assessed via sytox staining on flow cytometry or via acridine orange/propidium iodide staining on the automated cell counter. **e** Flow cytometric analysis of cell surface CD80, CD86 and HLA-DR expression on B cells before and after in vitro expansion, and with or without electroporation. Hashed bars indicate B cells electroporated with no mRNA, GFP mRNA or HPV16 E7 mRNA. ***, *p* < 0.001

In this experimental system, B cells to be used as APCs were isolated via positive magnetic selection from deidentified, cryopreserved HLA-A*02 positive healthy donor peripheral blood mononuclear cells (PBMC) from the NIH Clinical Center Blood Bank. PBMC from the same donor were retrovirally transduced with an HPV16 E7_11–19_-specific, HLA-A*02:01-restricted TCR to create an autologous system. Isolated B cells readily expand in culture ~ 50-fold over a seven-day period (Fig. 2a). These data suggested that B cells can be readily expanded as needed for desired experimental conditions.

### B cells were readily electroporated with mRNA and remain viable

Expanded autologous B cells were electroporated with mRNA encoding full length HPV 16 E7. As a positive control for electroporation efficiency, separate B cells were electroporated with mRNA encoding GFP and analyzed 24 h later by fluorescent imaging and flow cytometry for green fluorescent protein (GFP) fluorescence. Fluorescent imaging revealed GFP fluorescence in cells electroporated with GFP (Fig. 2b). Flow cytometric analyses demonstrated that > 90% of B cells express GFP following electroporation (Fig. 2c), indicating that electroporation of GFP mRNA can be used as a positive control indicating successful electroporation. High B cell viability was observed following electroporation without mRNA (control) or following electroporation with GFP mRNA or HPV16 E7 mRNA (Fig. 2d). Further, B cells expanded in culture expressed activation markers CD80, CD86 and HLA-DR to a greater degree than B cells that were not expanded. Expression of these activation markers did not significantly change following electroporation (Fig. 2e). These data suggest that ex vivo B cells can be electroporated with mRNA of interest and that electroporated B cells remain viable and express CD80/86 and HLA-DR required for TCR co-stimulation and cross presentation of intracellular antigen, respectively.

### Co-culture of antigen-loaded B cells with TCR engineered T cells allowed detection of antigen specific responses

**Fig. 3:**
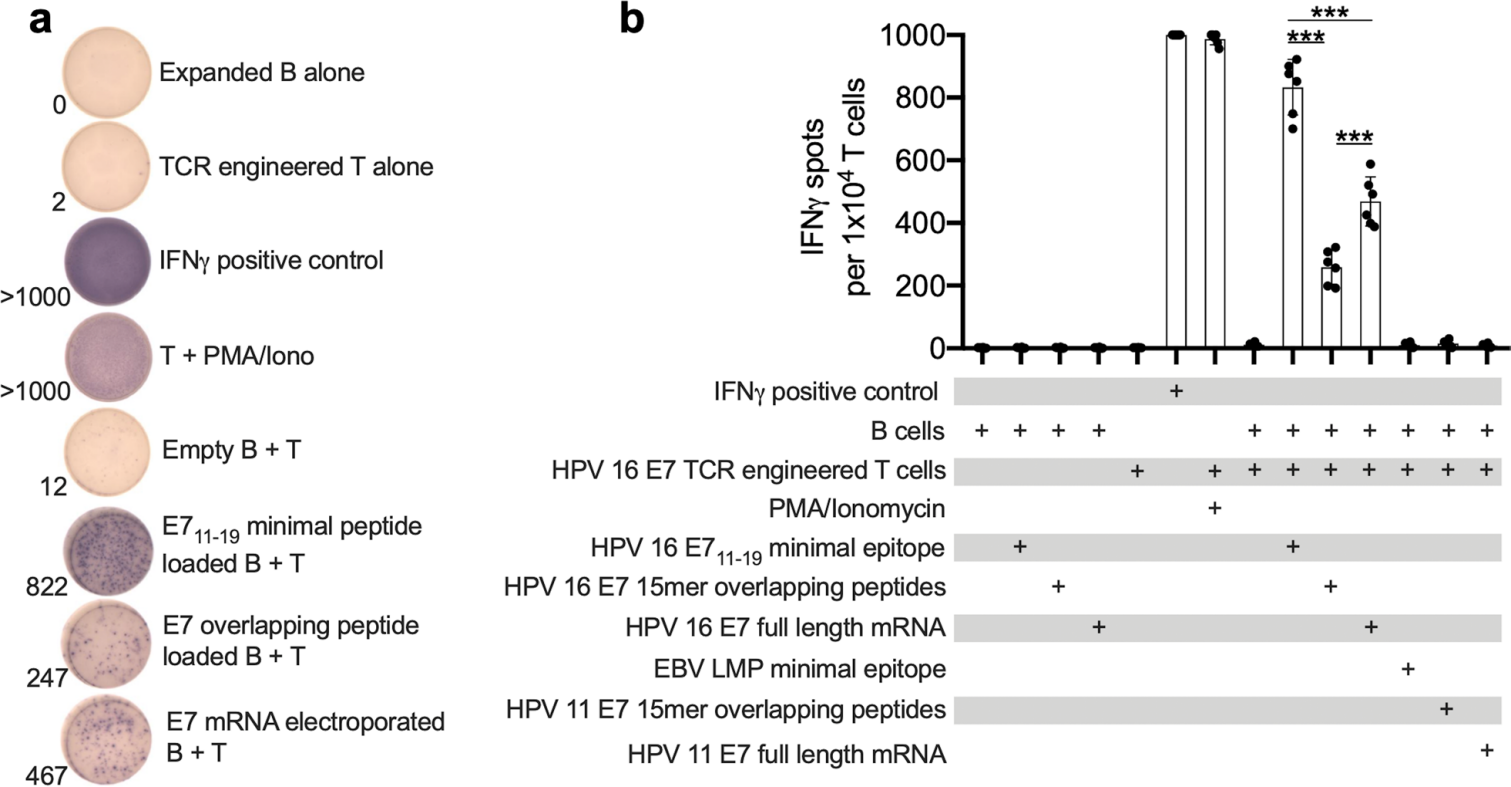
B cells naturally process and present minimal epitope from electroporated full-length mRNA. **a** Representative photographs of ELISpot wells resulting from co-culture of expanded autologous B cells loaded with E7_11–19_ minimal peptide or 15mer overlapping peptide spanning the entire E7 protein, electroporated with mRNA encoding full-length E7, or controls for each condition; **b** quantification of IFNγ spots from all experimental controls and conditions. Spot counts greater than or equal to 1000 are depicted as 1000. Representative results from one of three independent assays with similar results. ***, *p* < 0.001

ELISpot was used to detect antigen specific T cell responses upon co-culture with autologous B cells. As a model of antigen-specific T cells, CD8+ cells isolated from healthy donor PBMC were transduced to stably express an HPV16 E7-sepcific, HLA-A*02 restricted TCR. Co-culture of these TCR engineered T cells with B cells pulsed with E7_11–19_ minimal epitope, but not an irrelevant 9mer peptide from EBV LMP2, resulted in robust IFNγ production and a high spot count (Fig. 3a, b). Co-culture of TCR engineered T cells with B cells pulsed with the same concentration of 15mer overlapping peptide spanning HPV 16 E7, but not overlapping peptide spanning HPV 11 E7, resulted in IFNγ production but to a lesser degree than that observed with the minimal epitope. Finally, co-culture of TCR engineered T cells with B cells electroporated with mRNA encoding full length HPV 16 E7, but not mRNA encoding full length HPV 11 E7, resulted in robust IFNγ production and a high spot count. These data suggested that autologous B cells were able to efficiently process and present a known T cell antigen following electroporation with full length mRNA encoding the parental protein.

## Discussion

Processing and presentation of a T cell antigen from an endogenous intracellular full length protein is a complex multi-step process [9]. The key advantage of using APCs electroporated with mRNA encoding full protein as a candidate T cell antigen source is that any observed T cell responses are specific for an intracellular antigen that must be naturally processed and presented. This result indicates that the same antigen may be naturally processed and presented on cancer cells from the same patient. Loading APCs with exogenous peptides can lead to presentation of antigen on HLA class I and class II molecules, but such peptides may bypass one or more steps in antigen processing that is observed with antigen derived from an endogenous intracellular full-length protein [4, 10]. Doubrovina et al. identified 36 putative HLA-class I-restricted T lymphocyte epitopes using APCs pulsed with 15mer overlapping peptides spanning the parental protein. One quarter of these T lymphocyte responses were absent when APCs expressing the full-length endogenous protein were used instead of APC pulsed with 15mer peptides [8]. These data support that the repertoire of HLA class I-restricted T lymphocyte antigens derived from endogenously expressed intracellular protein is more restrictive than that derived from overlapping 15mer peptides, and that the use of 15mer overlapping peptide could lead to the identification of T cell antigens that are not naturally processed and presented from an intracellular full-length protein and thus clinically irrelevant.

Another potential pitfall of using APC pulsed with exogenous 15mer peptide solely to determine the presence of antigen-specific T cell responses is that the concentration of peptides that enter cells may be significantly greater than the concentration of peptides derived from natural processing and presentation of intracellular full-length protein. Competition for HLA pocket binding is well established [11, 12], and commonly used peptide concentrations in the micromolar range may skew the HLA class I peptidome toward pulsed peptide or displace low affinity but naturally processed and presented intracellular antigens. Electroporating mRNA encoding full length protein into cells may allow more physiologic levels of intracellular protein to be translated, processed, and loaded onto class I molecules for presentation to T cells. In our experiments, electroporation of mRNA encoding full length HPV 16 E7 led to greater activation of TCR engineered T cells upon co-culture compared to B cells pulsed with 15mer overlapping peptide spanning HPV 16 E7 but reduced activation compared to E7_11–19_ minimal epitope peptide. Greater activation of TCR engineered T cells with minimal epitope compared to 15mer overlapping peptide would be expected given that the 15mer peptide pool was loaded into B cells at the same concentration (0.1 µg/mL) but consists of 22 total 15mer peptides. However, these experimental conditions are not directly comparable as the quantity of antigenic material entering APCs under these different conditions in uncontrolled and 15mer peptides must be processed inside cells to a degree to be loaded onto HLA class I. These data simply confirm that CD40L-activated B cells can process and present a T cell antigen when pulsed with minimal epitope, 15mer overlapping peptide and when electroporated with mRNA.

The use of TCR engineered T cells as a model antigen-specific T cell may allow detection of greater magnitude responses compared to those observed from low-frequency, naturally occurring T cells. TCR engineered T cells were used in this work to demonstrate proof-of-principle responses against APCs loaded with peptide or electroporated with full length mRNA. Low frequency antigen-specific T cell clonotypes may need to be expanded with one or more rounds of in vitro stimulation before experimental detection can occur. The frequency of a T cell clonotype that required to detect responses upon co-culture with APC loaded with mRNA is unclear and required further determination.

Cells other than B cells can be used as APCs. Dendritic cells can be isolated or cultured and matured from PBMC but require high cell numbers to generate a reasonable number of cells for experimental use [13]. B cells are readily isolated by magnetic sorting, and readily expand upon exposure to CD40L and IL-4 to numbers that allow a large variety of experimental conditions [14–16]. Tumor cells may process and present antigen differently than professional APCs of hematopoietic origin. Use of B cells as APCs may not fully model how antigen-specific T cells would response to a tumor cell presenting cognate antigen, but rather simply serve as an experimental tool to determine the antigen-specificity of a given T cell clonotype.

When designing expression vectors for the mRNA encoding the full-length protein of interest, inclusion of trafficking or degradation signals proteins can shuttle protein toward HLA class I or II processing. This could allow detection of antigen-specific CD8 or CD4 T cells restricted to antigen presented on HLA class I or II, respectively. Whereas engineering HPV 16 E7 to express a ubiquitin tag would promote proteasomal degradation and initiation of antigen processing for the HLA class I pathway [17], we flanked our gene of interest (HPV 16 E7) with Signal F and DC-LAMP. Signal F is derived from the N-terminal of LAMP-1 and is a signal peptide used to target proteins to the endoplasmic reticulum, and DC-LAMP is a lysosome-associated membrane glycoprotein that contains a sorting signal that mediates lysosomal targeting and aids in the processing of antigen within phagosomes in the HLA class II pathway [18–20]. Though not experimentally determined here, these modifications may allow efficient processing and presentation of HLA class II-restricted antigens. Our experimental validation that the HPV 16 E7_11–19_ epitope is presented via HLA-A*02 while flanked with Signal F and DC-LAMP suggests that HLA class I-restricted epitopes are still naturally processed and presented from full length intracellular protein despite these lysosomal pathway-targeting signals. Both CD8+ and CD4+ T cells play important effector roles in anti-tumor immunity [21], and investigators interested in querying antigen-specific responses in pools containing both CD8+ and CD4+ T cells should consider inclusion of Signal F and DC-LAMP in mRNA expression constructs.

Potential limitations of using electroporated mRNA to mimic intracellular protein for the query of antigen-specific T cell responses are cost and technical considerations. Engineering expression vectors for each candidate protein of interest form which a T cell antigen may be derived can be costly. The experimental techniques involved in electroporation of autologous expanded APCs with in vitro transcribed mRNA are more complicated than pulsing APCs with 15mer overlapping peptides. A reasonable approach may be to use overlapping 15mer peptide to screen for positive antigen-specific T lymphocyte responses, with validation that responses are specific for naturally processed and presented antigen via electroporation of mRNA.

## Conclusions

In conclusion, the ability to accurately assess peripheral or tumor infiltrating T cells for antigen-specific responses is an important correlative readout for clinical trials. In cases where the antigenic minimal epitope or HLA restriction element are not known, the use of autologous cells as APCs ensures HLA compatibility, but the technique used to introduce protein from which the antigenic minimal epitope may be derived may influence experimental outcomes. The approach of electroporating mRNA encoding full-length protein into APCs ensures that any positive responses upon co-culture with T cell pools of interest are to naturally processed and presented antigens. This approach should be considered by investigators when it is important to ensure that experimentally observed T cell responses are to naturally processed and presented antigens.

## Methods

### NIH3T3-CD40L cell line

The NIH3T3-CD40L cell line, described previously [22], was cultured at 37 °C with 5% CO_2_ in DMEM media containing 10% heat inactivated human serum, 100 U/mL penicillin, 100 µg/mL streptomycin, 5 µg/mL gentamycin, 2 mM l-glutamine and 25 mM HEPES buffer.

### Purification and expansion of autologous B cells

B cells were isolated form PBMC via positive magnetic selection using CD19 microbeads per manufacturer recommendations on an autoMACS Pro Separator (Miltenyi Biotec). B cells (5 × 10^6^) were then co-cultured in a T-175 flask at 37 °C with 5% CO_2_ with irradiated (6000 rad) NIH3T3-CD40L feeder cells [14, 15] at a 1:1 ratio in 20 mL of complete B cell media consisting of IMDM, 10% heat inactivated human serum, 100 U/mL penicillin, 100 µg/mL streptomycin, 5 µg/mL gentamycin, 2 mM l-glutamine and 200 U/mL IL-4 [16]. Media was freshened on day 3 by adding an additional 10 mL of media. Expanded B cells were harvested and counted on day 7. If necessary, co-culture with NIH3T3-CD40L cells was repeated until B cells were expanded to numbers needed for experimental use. Cells were counted on a Nexcelom Cellometer Auto 2000 using acridine orange/propidium iodide per manufacturer recommendations.

### Generation of in vitro transcribed (IVT) mRNA

pcRNA2SL expression plasmids encoding GFP, HPV 16 E7 or HPV 11 E7, flanked by Signal F and DC-LAMP, were generated commercially (GenScript). Plasmid linearization was accomplished in a 6 µL reaction volume by combining 1 µg of plasmid (0.5 µg/µL) with SmartCut buffer and NotI HF restriction enzyme (New England Biolabs) per manufacturer recommendations. Following incubation at 37 °C overnight in an Applied Biosystems thermal cycler, the reaction mixture was heat inactivated for 20 min at 65 °C, followed by a 5-min incubation at 4 °C. mRNA was transcribed and polyadenylated from 1 µg of linearized expression construct using the mMESSAGE mMACHINE T7 ULTRA Transcription Kit per manufacturer instructions (Thermo Fisher Scientific). IVT mRNA was purified using the RNeasy Mini Kit (Qiagen).

### Electroporation of APCs

Eight µg of IVT GFP, HPV 16 E7 or HPV 11 E7 (control) mRNA was added to an electroporation cuvette containing 5 × 10^6^ expanded B cells (100 µL volume) in Opti-MEM and mixed gently by hand. Electroporation was performed using a BTX electroporator (BTX Harvard Apparatus; 150 V, 20 ms, 1 pulse). Electroporated B cells were immediately transferred to polypropylene tubes containing complete B cell media and housed at 37 °C with 5% CO_2_ until used experimentally.

### Peptide pulsing of APCs

Expanded B cells (4 × 10^5^ cells) were suspended in 1 mL complete B cell media and incubated with 0.1 µg/mL of either pooled 15mer (overlapping by 11 amino acids) peptides spanning HPV 16 E7 or 0.1 µg/mL of E7_11–19_ peptide at 37 °C with 5% CO_2_ for 1 h. B cells were washed in complete B lymphocyte media prior to co-culture with TCR engineered T cells. In some conditions, pooled 15mer overlapping peptides spanning HPV 11 E7 or a 9mer peptide derived from the Epstein-Barr Virus latent membrane protein 2 (EBV LMP) were used as controls.

### TCR engineered T cells

T cells engineered to express a previously characterized HPV16 E7_11–19_-specific, HLA-A*02:01-restricted TCR were generated as described [3]. Briefly, CD8+ T cells isolated from HLA-A*02 positive healthy donor PBMC via negative magnetic selection (Miltenyi Biotec) were retrovirally transduced with the HPV16 E7_11–19_-specific, HLA-A*02:01-restricted TCR and cryopreserved. A mouse constant region was incorporated into the retroviral expression vector, and flow cytometry was used to verify that > 90% of transduced T cells expressed the TCR of interest. Cryopreserved TCR engineered T cells were thawed and rested overnight in media consisting of 50% AIM V media, 50% RPMI media, 25 mM HEPES buffer, 5% heat inactivated human serum, 100 U/mL penicillin, 100 µg/mL streptomycin, 10 µg/mL gentamycin, 2 mM l-glutamine, 1.25 µg/mL Amphotericin B and 6000 U/mL IL-2 overnight prior to use.

### ELISpot

Electroporated, 15mer overlapping peptide, or minimal epitope peptide-pulsed B cells (2 × 10^4^) were co-cultured with TCR engineered T cells (1 × 10^4^) at a 2:1 ratio for 20 h at 37 °C with 5% CO_2_, and ELISpot was performed per manufacturer recommendations. The final volume of each well was 200 µL; 100 µL B cell media and 100 µL T cell media. Spot counts for ELISpot assays were measured on an Immunospot ELISpot plate reader (Cellular Technology).

### Flow cytometry and fluorescence microscopy

Freshly sorted or cultured B cells with or without electroporation were assessed for cell surface expression of surface protein markers using anti-human CD80 (clone 2D10), CD86 (IT2.2) and HLA-DR (L243) antibodies from Biolegend directly conjugated to fluorophores. Cells were suspended in 1X PBS containing 1% BSA. Non-viable cells were excluded with sytox blue. Data was acquired with FACSDiva software on a BD Fortessa LSRII analyzer. Data was analyzed using FlowJo (VX10.0.7r2). Photomicrographs of B cells electroporated with IVT mRNA encoding GFP were obtained on an EVOS imaging system (Thermo).

### Statistics

Comparison of multiple sets of data was achieved with one-way analysis of variance (ANOVA). Analysis was performed using GraphPad Prism v8.4.1.

## Data Availability

All data supporting the results of this work are included in the manuscript. All materials are available upon reasonable request to the corresponding author.

## Abbreviations

APC: Antigen processing cells
GFP: Green fluorescent protein
HLA: Human leukocyte antigen
PBMC: Peripheral blood mononuclear cells
TCR: T cell receptor
